# Canon Ainger's True Physician

**Published:** 1894-08-11

**Authors:** 


### The Hospital

An Institutional Journal of

The Medical Sciences and Hospital Administration, with

AN EXTRA NURSING SUPPLEMENT.

Vol. XVI.—No. 411.] [Aug. 11, 1894.

### CANON AINGER'S TRUE PHYSICIAN.

The Rev. Canon Ainger, the natural, as he is the actual, successor to Dr. Vaughan in the Mastership of the Temple, is a typical representative of one aspect of the culture of the closing years of the century. The British Medical Association, which always inaugurates its annual sessions by a public religious service, this year made choice of Canon Ainger for its preacher at the Bristol Congress; a discriminating choice. The preacher selected as the subject of his prelections, "The Method of the True Physician."

It is more than interesting, it is advantageous for us to see ourselves in the mirror of another man's mind. It may be advantageous in a high degree if the mind be that of a judicious critic, and at the same time of a sympathetic friend. To the body of the medical profession Canon Ainger has approved himself in both these capacities. In the mind of the true Churchman there is one standard of the sublime and the excellent. Canon Ainger at once raised the physician to the highest possible degree of exaltation by instituting a comparison between him and the exalted Person who has been the world's standard of all that is great for close upon two thousand years.

The "Method of the True Physician" was the subject of Canon Ainger's thoughts; the "method," not the methods. There was no intention of setting before his audience a school lesson. The object was to show the unity of many-sided truth and work in the one great vital method of searching for the true and doing the right. A simple incident furnished the preacher with an apt illustration for his theme. A poor woman, diseased with an issue of blood for twelve years, hearing of great wonders done by a striking Personality, and probably having herself watched and noted that striking Person for many weeks or months, suddenly concludes that He is worthy of confidence, and that she will at least trust herself to His skill. There was no superstition, Canon Ainger contends, in the woman's mind, but a reasonable faith. She, according to her lights, recognised in Jesus the possession of knowledge and power. To knowledge and power, therefore, she gave herself up, not to magical arts and the pretence of power. Assuming that Jesus was what the Church has always declared Him to be, the incarnation of the All-knowing and the Almighty, it is evident there was no superstition, but a reasonable faith.

Canon Ainger sees in modern medicine a foe to superstition; and not to superstition only, but to poor work, to low aims, to everything which is not severely true and innately right. The one great aim of scientific medicine is to search and examine until the truth and inwardness of ill-health is found, and to establish treatment upon that. This is mostly a slow process; it can seldom be a quick one. "Cures," "specifics," "short cuts" Canon Ainger suspects. The unscientific world on the other hand loves "specifics," "short cuts," "cures," and will have them. This is an intellectual deficiency, the preacher tells us, but it is a moral deficiency still more. The seeker after "specifics" is a kind of gambler; he wants to obtain a result without paying the price which nature herself has put upon the result. That cannot be. At heart the man who wishes to be treated by "cures" and "specifics" is as immoral as the man who trades in them for pecuniary profit. The whole thing is a fraudulent attempt to set aside the irrevocable laws of nature, which in terms of Canon Ainger's life conclusions are the expressions of the unalterable will of God.

There is a deep insight into the thing that is true here; into the ultimate truth, which always is unity. Canon Ainger makes for us a comparison between true medicine and true religion; and conversely between medical quackery and spiritual quackery. There are many persons, he says, who do not see the thing that is true in religion; who do not recognize the nature of spiritual pathological processes; who do not, or will not understand that the therapeutics of the soul must work harmoniously with spiritual physiology, and that only by so working can manly, vigorous, and permanent spiritual health be restored. The spiritual quack dearly loves "specifics," "cures," "short cuts," and his patients love these things quite as much, and so a brisk trade is done, and the world is drugged and its progress hindered; and neither moral nor material health and beauty can be made possible on an extensive scale.

Perhaps we should search in vain for quite such utterances as these in the religious literature of any periods earlier than the latter half of the nineteenth century. Canon Ainger makes it very clear that he has become familiar with the methods and aims of science, particularly of medical science. He seems to us to have profited much, both intellectually and morally, by his investigations in scientific fields. He is minded also to pay back some of the debts he feels he owes to the men who have made modern medical science what it is. The medical profession, he tells us, has done noble work in the purification of the intellectual and moral atmospheres, as well as in the sweetening of the material. When it insists upon discovering things as they are, when it refuses to accept first appearances, when it forces its way at all risks to the very roots and minutest rootlets and tendrils of the true facts, it is setting a noble object lesson to the spiritual physician. When it aims at healing disease from the very lowest depths of its pathological processes, and will by no means be content until the sick man is not only convalescent but well, not only well but strong and perfect in every function of his body, it again sets before the spiritual physician a mark and a standard than which no lower should be his aim. In truth, medicine as we see it, and as Canon Ainger sees it, is, after Christianity, the greatest moral purifier of the times. Truth, absolute truth; and perfectness, absolute perfectness, are the Alpha and Omega of its life.

It must give a medical man pause when he perceives how entirely Canon Ainger is at home in the essential science of medicine, and yet how fully and implicitly he accepts the conclusions of the Church in things moral and spiritual. Is it true, as this cultured Churchman of the close of the century believes, that in essence the soul of Christianity and the soul of science and scientific medicine are one and the same? They both aim at the thing as it is, and will be content with nothing less. It is something for us to think of that a man like Canon Ainger believes it to be true. The philosophic doctor does not see in his science a truth isolated and independent of all other truth. He sees in the facts, and especially in the one unifying fact of his science, the fact of life, a part of the one universal and all-pervading truth of the world. The doctor aims at the centre and soul of all things; at the true essence and cause of all causes and effects; at unity. The divine, working from another standpoint, has an identical aim. Is there a centre at which they and all other truth-seekers will ultimately meet? Canon Ainger thinks there is, and we agree with him. It will be a good day for the world when all its physicians, alike of the body and the soul, cease from their prejudices, and seek with the candle of perfect simplicity for the simple truth.

